# Chit-Chat

**Published:** 1874-01

**Authors:** 


### Editorial Chit-Chat.

Our New Place.—Since the last issue of the Bistoury, we have transferred our office to the Surgical Institute on Hudson Street, where we are housed comfortably, and much more conveniently, with our patients. We are happy to announce that, although it has been but three weeks since we opened this institution, yet, every private room is occupied by patients, and the free wards have been almost as well patronized. We well knew that such a retreat was needed in Elmira, but scarcely expected to fill it quite so soon.

That our readers may be able to judge of the character of our building, we beg leave to publish the following description of our institution, taken from the *Daily Advertiser* of this city ; at the same time asking pardon for exhibiting this bit of egotism. Our readers are well aware that we very rarely, or never, encumber our columns with any matter relating to ourself or our business; they will therefore the more willingly pardon us for this intrusion, particularly as it will give them an excellent idea of the sort of place the Bistoury is issued from. And now for the article :—

"Elmira Surgical Institute. — Dr. Up deGraff has done for himself and this community a good thing in the establishment of his surgical institute, in the Fifth Ward. It is an institution, the need of which has been frequently felt in our midst, there being no proper place where could be conveyed and kept, those who might meet with accident to limb or body.

"The Institute is very pleasantly situated on the south side of Hudson street, just west of the railroad. All about that neighborhood there are many fine, large trees, especially in the immediate vicinity of the building. It was formerly the school house of that district, and much pains had been taken to surround it with fine shade trees. The lot on which the Institute stands is one hundred and twenty feet front by one hundred and fifty-five feet deep, and the building itself, outside of everything, on the ground, is fifty by seventy-five feet. It is but two stories in height, but the distance is so great between the floor and the ceiling of each, that the whole structure is a very prominent object amidst the pleasant dwelling houses of that neighborhood. It is surmounted by a cupola twelve or fourteen feet square, from the roof of which rises a thirty-five foot pole from which flies, as occasion may warrant, a hospital flag of the regulation size and color, and the only one that has ever flown in this valley.

"A balcony, wide and roomy, runs around three sides of the building, in both stories, and in summer time will form a very comfortable promenade for the patients, or place where convalescents, unable to walk, can be wheeled.

"The front of the building looks to the north. You enter through a doorway, more than ordinarily large, and are ushered into a hall of goodly proportion, being eight feet wide and forty feet long, with ceiling twelve feet and a half high. Immediately to the right, is the operating room, lit by large windows and fitted up with all the latest apparatus for surgical operations. To the rear of this, is Dr. UpdeGraff's private office, one of the cosiest apartments imaginable, provided with easy chairs and elegant furniture, showing the good taste and judgment of the proprietor, and, as well, his love of genuine comfort. From this room the Bistoury, which speaks to its twenty thousand readers every quarter of a year, takes its rise and flows on in its uninterrupted course of prosperity. Beside this office is the opthalmoscope room, and in the rear of both, the matron's apartment, twenty-five by thirty feet, and in the rear of that, a large, well arranged and convenient kitchen.

"On the east side of the hall are the parlor or reception room, three rooms for patients, and a large dining hall twenty-two by thirty-five feet, and also the apartments of Dr. Theo. N. Lescher, the accomplished assistant surgeon, and resident physician of the institution.

"On the second floor front there are five bed-rooms, to the rear of which is a large ward capable of accommodating twenty-five patients. There are, besides, on this floor, two other wards, one of which will accommodate eight, and the other fifteen patients. Off the rear hall there are six private bed-rooms.

"The appointments of the Institute are all of the most complete nature, and the most suitable for the purposes for which they are intended.

"On Wednesday evening of this week the building will be thrown open to the public, and the Doctor will be "at home" to receive those of his friends who may feel inclined to examine the Institute and congratulate him upon the inauguration of an enterprise to which he has been looking for many a year."

And now, having waded through this, perhaps you are better prepared to digest the following account of our "opening," as related by the editor of the *Gazette*. In this way, it will be seen, that we not only succeed in inflicting our readers with a wholesome puff of ourself and institution, but also fill our space allotted to chit-chat with comparatively little effort upon our part. Perhaps in this we are wise, or otherwise, but you can best judge by reading what follows:

Elmira Surgical Institute—Formal Opening Last Evening—A Superb Season of Sociality and Speech-Making.—The reception given by Dr. Up de Graff to his many friends, at his new Surgical Institute last evening, was on a scale of magnificence and liberality fully in keeping with that exhibited by the energetic head of the establishment in finishing and furnishing of the building. The old school building would not be recognized except by the location, so changed and improved is its appearance. The building is divided up into convenient wards, parlors, reception rooms, operating rooms, dining room, &c., all finely furnished. The beds and cots are all of the best quality, and every room in the building is pleasant and cheerful. There is a wide balcony to both stories on three sides of the building, from which a fine view is obtained. The formal opening was a very brilliant affair. There were a large number present from a distance, among them President Packer of the Lehigh Valley R. R. By nine P. M., upwards of one hundred ladies and gentlemen were present.

"Dr. Up de Graff and assistants were everywhere present, showing the guests about the building and explaining everything connected therewith. No effort was spared to make the occasion one of great pleasure to all present. About ten o'clock the dining-room doors were thrown open, where two long tables had been set bountifully supplied with every desirable luxury. Rev. Thos. K. Beecher occupied the honored position at the head of one table, and Mayor Caldwell the other.

"In an appropriate and happy speech, Mayor Caldwell proposed the health of Dr. Von-Moschzisker, who responded in eloquent terms. His remarks were highly and deservedly complimentary to the energy and skill of Dr. Up de Graff. In closing, he proposed the health of *Doctor* Beecher, who in response made one of his happy and graceful speeches, in which he referred to the public spirit and enterprise of the city, and especially to the public spirit exhibited by Dr. Up de Graff, not only in this enterprise, but in all others calculated to advance the prosperity of the city. He spoke in high terms of the personal worth of the host, his honesty and integrity, and was happy to recognize him as his friend. He proposed the health of Dr. Up de Graff. The Doctor briefly responded and stated that the Institute was not a purely charitable one; while the poor will never be turned away and will be provided for, those who are able to pay will be charged accordingly, but there will be no difference in the care and attention bestowed.

"Secretary Youmans, of the Board of Education, gave an interesting history of the old school building, and predicted great success in the enterprise just inaugurated.

"Mr. Packer was also called out. He wished to bear testimony to the professional and personal worth of his friend, the Doctor. * *
* * * * * * *

"The opening of Elmira's first hospital was a grand success, and an occasion that will long be remembered by those who enjoyed the hospitalities of the liberal-hearted doctor."

This article, we are sorry to say, is not as well written as it would have been had *we* had a hand in it. We think the "doctor" is entirely too much praised, while the distinguished guests present, and the part they took in the entertainment, were overlooked. And now, if our readers do not know "all about us" and our place of business, it certainly cannot be by reason of any fault of ours.

---

"H."—The confusion attending the removal of our headquarters, and the uncertainty of our whereabouts, for the past three months, has separated us from our joking friend, who fails to give us his customary contribution in this department of the Bistoury. We hope he will become so familiarized with his new lounging place as to be able to present his budget in season for our next number.

"Once a Month."—A neat little monthly journal, published by Robert M. Watts. Robert is recognized in this community as an excellent job printer, and his journal evinces the further evidence of his ability as a local editor.

---

Which?—The trial of a noted New York criminal has revealed his name to be Genet; but, pending his sentence, it was mysteriously, and without consent of court, changed to Genot. 'Tis said he has embarked in his yacht, but the newsboys declare it was a clipper.

---

Spain.—We are tired, as doubtless are most of the reading public, of the display made in the telegraphic columns over the Virginius. We are glad the affair is settled, and that our government did not allow Spain to Cast-a-leer at the United States, without apologizing for the insult.

---

Penn. vs. N. Y.—For some time, a direful encounter has been going on in the Surgical Institute. The war has waxed hot over a contest of skill in the queer, and never to be fully comprehended game, of "back gammon." The contestants are a lady, one from a Pennsylvania city, and a gentleman, an ex-minister, from a city in the Empire State. The minister has secured *one,*—mind you, only one little game, thus far, while the lady has captured all the rest. 'Tis strange, but Otis so.

---

American Wines.—Our American champagnes are fast becoming popular with our people, and deservedly so. We observe that almost every hotel of importance gives a place to these champagnes upon their wine lists, there being quite as much a demand for them as for the foreign article. When you have occasion to drink pure and unadulterated wines, seek the Hammondsport variety, and order through Lormore Bros. & Co., of this city, who are the agents for both the "Urbana" and "Pleasant Valley" Wine Companies, and from whom you can procure the goods at the same rates as at the cellars. The Messrs. Lormore Bros. & Co. are constantly shipping their champagnes to all parts of the United States, attesting the appreciation our people have of our productions.

A New Tenor.—We have a new tenor in our city—a startling one—one that can always astonish the natives with the melody of his voice. He made his *debut* recently in the hall of the Surgical Institute, and succeeded in astonishing the large audience there assembled to welcome him to our musical boards. He appeared to best advantage in the song of "One Fish Ball," a musical extravaganza, which was rendered in a manner that startled and amazed all who had the good fortune of listening. It is believed that the artist will not again appear in public until next year, by reason of the severe strain his "vocal chords" were subjected to in rendering that musical word ba-*a*-all.

---

In The Nick o' Time.—We expressed our fears in another column, that "H." would not put in an appearance to relate his customary joke in season for this number. But late last evening, he came, looked suspiciously at the roll of "proof" lying upon our table, and innocently inquired whether Bistoury was out. Being assured that the printer had measured the "matter" and said it was "full," our friend seemed easy in mind, and thought it safe to chat away in his usual vein, without danger of appearing in print. But this morning, behold, John comes hastily to our sanctum, with the startling demand for more "copy," and we seize the opportunity to give "H's" latest joke.

Years ago when H. was in the dry-goods business, a seedy looking individual appeared at his counter, the day before Christmas, and desired to look at a scarf,—a cheap one, he said, one out of style, an "old residenter," in fact, as he wanted it for a present, and couldn't afford to "go much on it." H. looked among his old and discarded stock, and succeeded in finding what he supposed would meet the requirements in the case, and brought forth the article for the inspection of the seeker after cheap gifts. It had a cost mark upon one corner, as is the usual form with merchants, and this particular mark happened to read "B C." Seedy examined the scarf a moment, then glanced at the tag containing the mark, and removing a quid of tobacco from his mouth, exclaimed: "Say, stranger, can't you give me something just a *little* more modern, even if it costs more! Something in Anno Domini for instance, as my gal might not like the style as old as B. C!"

A Centenarian.—There is an old colored woman in this city, who has been in the employ of the same family for more than twenty-five years, and who is still making herself useful, at the age of one hundred and eight.

---

In Welsh.—We have a couple of lady patients in the Institute who insist upon addressing us in their native tongue,—Welsh. The language is such as to impress us with the belief that they are swearing at us, but being ministers' wives, and most estimable ladies, of course we cannot harbor such a thought for an instant.

---

The Envelope.—Look out for it! It is in this number, and will answer for you to enclose your subscription in. Fill the blank, enclose the money, and do not forget your arrearages, if you owe any. Pay promptly, we can then better afford to give you an entertaining, useful and sprightly Journal.

---

Precaution.—Ever own a musical box? They are elegant means of enjoyment, and a never ceasing amusement for the baby. Some times they require cleaning and oiling,—the musical box is meant, not the baby—and when that moment arrives, don't try to do it yourself, if you do, it is likely to cost you from fifteen to twenty-five dollars for the experiment. We speak from experience, possessing, therefore, peculiar qualifications as an advisor upon the subject mentioned. If you conclude, as we did, that you are as capable of cleaning the machine as your jeweler, be sure that it has "run down"—*entirely* and completely down,—don't allow the least force of the spring to exert its influence upon the cylinder, else you will hear such a racket, when you come to detach the "fly wheel," as will cause your hair to stand on end, unless perchance, you are bald headed. That marvelous combination of confusion, (worse confound-it, by reason of the cost incurred,) still rings in our ears; and, if baby and the balance of our innocent household, ever regain sufficient confidence in the instrument, to allow them to remain anywhere about the premises when their indulgent father is about to "wind up the machine," we shall have gained a place in their affection that, at this writing, seems to be entirely destroyed, when we are within reach of the crank of that agonizing box.

